# Dysregulation of TSP2-Rac1-WAVE2 axis in diabetic cells leads to cytoskeletal disorganization, increased cell stiffness, and dysfunction

**DOI:** 10.1038/s41598-022-26337-1

**Published:** 2022-12-28

**Authors:** Hao Xing, Yaqing Huang, Britta H. Kunkemoeller, Peter J. Dahl, Ohvia Muraleetharan, Nikhil S. Malvankar, Michael P. Murrell, Themis R. Kyriakides

**Affiliations:** 1 Department of Biomedical Engineering, Yale University, New Haven, USA; 2 Department of Pathology, Yale University, New Haven, USA; 3 Department of Molecular Biophysics and Biochemistry, Yale University, New Haven, USA; 4 Department of Physics, Yale University, New Haven, USA; 5 Systems Biology Institute, Yale University, New Haven, USA; 6 Microbial Sciences Institute, Yale University, New Haven, USA; 7 Vascular Biology and Therapeutics Program, Yale University, New Haven, USA

**Keywords:** Cell biology, Molecular biology, Endocrinology

## Abstract

Fibroblasts are a major cell population that perform critical functions in the wound healing process. In response to injury, they proliferate and migrate into the wound space, engaging in extracellular matrix (ECM) production, remodeling, and contraction. However, there is limited knowledge of how fibroblast functions are altered in diabetes. To address this gap, several state-of-the-art microscopy techniques were employed to investigate morphology, migration, ECM production, 2D traction, 3D contraction, and cell stiffness. Analysis of cell-derived matrix (CDM) revealed that diabetic fibroblasts produce thickened and less porous ECM that hindered migration of normal fibroblasts. In addition, diabetic fibroblasts were found to lose spindle-like shape, migrate slower, generate less traction force, exert limited 3D contractility, and have increased cell stiffness. These changes were due, in part, to a decreased level of active Rac1 and a lack of co-localization between F-actin and Waskott-Aldrich syndrome protein family verprolin homologous protein 2 (WAVE2). Interestingly, deletion of thrombospondin-2 (TSP2) in diabetic fibroblasts rescued these phenotypes and restored normal levels of active Rac1 and WAVE2-F-actin co-localization. These results provide a comprehensive view of the extent of diabetic fibroblast dysfunction, highlighting the regulatory role of the TSP2-Rac1-WAVE2-actin axis, and describing a new function of TSP2 in regulating cytoskeleton organization.

## Introduction

Diabetes mellitus is a leading cause of disability, cardiovascular disease, and mortality. The prevalence of diabetes in the US has increased from 9.8 to 14.3% over the past two decades^1^. All forms of diabetes are characterized by high blood glucose levels and are often accompanied by comorbidities such as hypertension or hyperlipidemia. Care for diabetic patients directly accounts for more than 24% of all US health care spending and results in more than $90 billion indirect cost to the economy^2^. Hence, there is an urgent need to better understand the disease and its complications in order to provide effective and affordable treatments.

Chronic wounds are a major complication associated with diabetes. Often presented as lower-extremity ulcers, diabetic wounds increase the risk of sepsis and amputation, having a tremendous impact on patients’ quality of life and the overall healthcare system^3^. Numerous biological processes are reported to be altered in diabetic wounds. On a molecular level, the expressions of several growth factors such as vascular endothelial growth factor (VEGF) and tumor necrosis factor alpha (TNF-α) are altered^4,5^. In addition, matrix metalloproteinases (MMPs)-1, -2, -8, and -9 are increased in diabetic wounds resulting in multiple ECM changes^6^. For example, collagen I and III, have irregular deposition^7,8^, and ECM maturation is delayed in diabetic wounds^9,10^. On a cellular level, diabetic macrophages lack temporal coordination during the wound healing process and exhibit a proinflammatory and profibrotic phenotype^11,12^. Diabetic endothelial dysfunction is associated with reduced nitric oxide production, decreased endothelial cell migration, and increased production of reactive oxygen species^5^. Moreover, keratinocytes and fibroblasts decrease their ability to migrate and proliferate under diabetic condition^13^.

Dermal fibroblasts are a population of ECM-producing cells with an active role in the wound healing process^14^. They not only deposit ECM to remodel the surrounding tissue, but also migrate to the injury site, amplify inflammatory signals, and contract the wound bed in an orchestrated fashion with other cell types^15^. As mentioned above, under diabetic conditions, fibroblast behaviors and ECM-production are compromised. Recent multiomics studies have highlighted both the temporal and spatial shifts in fibroblasts during wound healing^15^. By using single cell sequencing analysis of human diabetic foot ulcer cells, two independent studies have found a diabetes-specific fibroblast subpopulation that was enriched for collagen expression, including COL1A1, COL3A1, COL6A1^16^, and COL7A1^17^. In addition, YAP/ECM and focal adhesions-PI3K-Akt pathways were identified by pathway enrichment analysis, suggesting that mechanosensing might play a critical role in diabetic fibroblasts^16^.

TSP2, a matricellular protein, modulates cell behaviors by interacting with growth factors, ECM, and cell surface proteins through its various binding domains^18^. TSP2 plays an important role in skin homeostasis and wound healing. In TSP2-null mice, though wound re-epithelization rate was similar to control animals, they exhibited faster dermal closure and higher initial vascular density associated with increased MMP2 and soluble VEGF^19^. In addition, TSP2 expression was increased in mice with genetic deletion of either endothelial nitric oxide synthase (eNOS) or Akt1, and both models displayed delayed wound healing^20,21^. Importantly, deleting TSP2 in these models resulted in restoration of normal wound healing. TSP2 is predominantly secreted by fibroblasts, and in Akt1-null cells, TSP2 expression was increased and associated with lower levels of Rac1 activation. In contrast, TSP2-null cells and double Akt1/TSP-2 null expressed higher levels of active Rac1 suggesting that TSP2 inhibited Rac1 activation and relevant cell fibroblasts functions^21^.

TSP2 also directly influences matrix assembly. Specifically, TSP2-null mice expressed low lysyl oxidase (LOX) levels in their skins resulting in increased solubility of fibrillar collagen^22^. TSP2-null ECM displayed disorganized collagen fibers and was resistant to thrombosis via impaired von Willebrand factor adhesion^23^. Consistent with these observations, decellularized biomaterials from TSP2-null animals have been used successfully as small vessel grafts to reduce thrombosis^24^ and dermal wound dressings to accelerate wound healing in diabetic mice^25^. In the latter application, TSP2-null ECM induced altered ECM remodeling, and increased fibroblast recruitment and vessel maturation.

The homozygous leptin receptor deficient mouse model (db/db) has the hallmarks of adult diabetes mellitus such as obesity, insulin resistance, hyperglycemia, and hyperlipidemia^26^. It has been widely used as a model to study diabetic wound healing because it is delayed and displays many of the pathological processes observed in human wounds^27^. Despite the significant overlap, the model is limited in that it shows no difference from WT when wounds are splinted^28^ and the associated etiology does not exactly recapitulate the human disease^29^. Nevertheless, there is prominent cell dysfunction that can be probed to investigate relevant mechanisms affected by diabetes. A recent study has demonstrated elevated TSP2 levels in both diabetic human skin and db/db (DB) mouse skin, and delayed wound healing in DB model was strongly associated with increased TSP2 in the wound ECM^30^. Importantly, depletion of TSP2 in this model to generate a db/db TSP2 knock-out (DKO) revealed a normal wound healing phenotype without changes in the metabolic profile associated with diabetes. Consistent with this observation, the deletion of TSP2 in diabetic fibroblasts restored cell migration in a scratch assay. Mechanistic experiments showed that the supraphysiological level of TSP2 was regulated via the hexosamine pathway and, in part, by nuclear factor kappa B (NF-κB) pathway^30^.

Taken together, these studies highlighted the importance of fibroblasts and ECM expression in restoring tissue homeostasis after injury. However, the complete impact of diabetes on function of fibroblasts including their biomechanical properties remains poorly characterized.

In this study, we employed multiple state-of-the-art microscopy techniques combined with biochemical assays to provide a holistic biomechanical view of the dysfunctional state of DB fibroblasts. We discovered that DB fibroblasts displayed a more rounded shape than their wild type (WT) counterparts and deposited thicker ECM fibrils that hindered NIH/3T3 cell migration. In addition, they displayed multiple functional defects including lower traction force generation, 3D contractility, and migration and had increased stiffness measured by atomic force microscopy (AFM). These defects were accompanied by reduced active Rac1 and disrupted localization of WAVE2. In addition, we investigated the phenotype of dermal fibroblasts from DKO and found that it was similar to WT, which was consistent with a critical role of increased TSP2 in diabetes. Importantly, the findings identify a previously unidentified link between diabetes and irregular cytoskeletal organization in fibroblasts.

## Materials and methods

### Animals

c57bl/6J, db/db, and db/db TSP2KO aged 12–14 weeks were used. C57bl/6J and db/+ mice were purchased from Jackson Laboratory. The generation of db/db and db/db TSP2KO animals was described previously^30^. All animal studies were performed based on procedures approved by the Yale Institutional Animal Care and Use Committee (IACUC). All authors complied with the ARRIVE guidelines.

### Dermal fibroblast isolation and cell culture

Dermal fibroblasts were isolated from mouse dorsal skin as described previously^31^. Briefly, mouse dorsal skins were shaved, excised, and incubated in 25 μg/ml Trypsin (Sigma) overnight at 4 °C, and dermis was then separated and digested using collagenase IV (Worthington Biochemical). Cells were then pelleted, resuspended, and plated in high glucose Dulbecco’s Modified Eagle Medium (DMEM, Gibco) with 10% (v/v) fetal bovine serum (Peak Serum Inc), 1% (v/v) penicillin–streptomycin (Gibco), and used between passage 1 and 2. NIH/3T3 (ATCC) were also maintained in high glucose DMEM with 10% FBS. High glucose DMEM media with 0.1% (v/v) fetal bovine serum (Peak Serum Inc), and 1% (v/v) penicillin–streptomycin (Gibco) was used as starving media. All cells were maintained at 37 °C with 5% CO_2_.

### Immunofluorescence (IF) staining and imaging

Cell-derived ECMs and cells were fixed for 30 min using 4% paraformaldehyde (ChemCruz). Cells were permeabilized using 0.1% Triton X-100 for 10 min. Both ECMs and cells were washed extensively using Phosphate-Buffered Saline (PBS, Gibco), and blocked using 1% BSA (Sigma-Aldrich) in PBS. Samples were incubated overnight at 4 °C with primary antibody, followed by extensive washing and secondary antibody incubation at RT for 30 min. To detect actin and nuclei, cells were incubated in 1:250 Phalloidin (Invitrogen) and 1:1000 DAPI (Invitrogen), respectively, in PBS at RT for 30 min. Samples were mounted using Vectashield antifade mounting medium (Vector Laboratories) prior to imaging. Fluorescent images were taken using a Zeiss Axiovert Fluorescence microscope. Confocal images were taken on a Nikon SP-5 spinning disk confocal microscope.

### 3D collagen gel contraction

600 μl of 1.3 mg/ml collagen I gel with neutral pH were prepared using sterile 1 M NaOH (Macron), 5X DEME (Gibco), rat tail collagen I (Corning), and water. 10,000 fibroblasts were suspended in 200 μl of media and added to the pre-gel mixture to reach a final collagen I concentration of 1 mg/ml. The resulting solution was added to 24 well plate coated with agarose (American Bio). The gel was polymerized at 37 °C for 30 min then separated from the wall of the well using a scalpel to allow contraction. 500 μl of media was added to each well. Gels were visualized using a high-resolution scanner (HP) at 12, 24, 48, and 72 h. Rate of contraction was quantified using ImageJ.

### 2D traction on polyacrylamide gel by traction force microscopy (TFM)

To measure the mechanical strain energy of primary dermal fibroblasts, traction force microscopy was performed on cells seeded on 12 kPa polyacrylamide gel coated with collagen as described previously^32^. Briefly, 12 kPa polyacrylamide gels were prepared using 12% polyacrylamide and 0.085% bis-acrylamide (Bio-Rad) with 1% 40 nm beads (Molecular Probes). The gels were polymerized using 0.05% w/v ammonium persulfate on an activated coverslip for 30 min at room temperature. 1 mg/ml of collagen I was then coupled onto the surface of the gel using EDC/EHS chemistry. Primary fibroblasts were plated onto the gel, washed after 4 h to remove unbound cells, and imaged immediately. A 60 × oil-immersion objective was used to image both the force-loaded images and cell-free images. Images were registered using ImageJ. The strain energy was calculated using custom-written code from Schwarz Lab^33^. Strain energy (W) was calculated, using $W= \frac{1}{2}\int F\cdot x dxdy$, where F was the local force, and x was the local displacement. The strain energy density was calculated as the ratio of strain energy and cell area.

### Cell-derived matrix (CDM)

CDM was prepared as described previously^24^. Briefly, confluent primary dermal fibroblasts were treated with 100 μM ascorbic acid (Alfa Aesar), replenished every 2 days. Gentle decellularization was performed on Day 7 using 40 mM NH_4_OH (J.T.Baker) and 0.5% Triton X-100 (American Bio) for 45 s followed by extensive washes with PBS. 2000 units/ml DNase I (Sigma-Aldrich) was used to treat the ECM for 1 h at 37 °C followed by extensive washes. The retained ECM was used for subsequent studies.

### Scanning electron microscopy (SEM)

SEM samples were prepared as described previously^34^. Briefly, samples were fixed in 2.5% paraformaldehyde in 0.1 cabodylate buffer (Electron Microscopy Sciences), dehydrated using an ethanol gradient, incubated in hexamethyldisilazane (Electron Microscopy Sciences), and air-dried overnight. Samples were then coated with 8 nm thick iridium using a sputtering tool (Cressington 208) and imaged using a Hitachi SU-70 scanning electron microscope.

### Transwell migration assay

Primary dermal fibroblasts were starved in 0.1% FBS media overnight. 8 μm pore transwells (Corning) were incubated in 50 μg/ml collagen I solution overnight at 4 °C and washed extensively using PBS the next day. 50,000 cells were plated on top of the tranwell in 100 μl of starving media, and 600 μl of regular media was added to the bottom of the transwell. Cells were allowed to transmigrate for 8 h, then fixed in 100% methanol, stained using diff-quick stain (Electron Microscopy Sciences), and imaged using a 20 × objective on a Zeiss Axiovert microscope.

For ECM coated transwell migration assay, the transwells were seeded with 50,000 primary dermal fibroblasts to produce cell-derived ECM as described previously^25^. Then, the standard transmigration described above using NIH/3T3 cells was used to study the effect of different ECMs on cell transmigration.

### Proliferation assay

10,000 primary dermal fibroblasts were seeded onto 96 well plates, incubated for 24 h, washed with PBS, and replenished with fresh media containing 0.5 mg/ml MTT (Thermo Fisher). 4 h later, MTT-containing media was removed, DMSO was added to each well, and absorbance was measured at 570 nm wavelength.

### Flow cytometry

A 200,000 cell suspension was incubated with anti-β1 integrin antibody and isotype control on ice for 30 min, washed extensively with DMEM, and incubated with APC-streptavidin for another 30 min. After extensive washing, cells were resuspended in PBS, and the surface expression of β1 integrin was measured using a 640 nm laser on a BD LSRII flow cytometer with appropriate size gating.

### Atomic force microscopy (AFM)

12 mm diameter coverslips were coated nonspecifically with 1 mg/ml collagen I solution at 4 °C overnight and then washed extensively with PBS. 5000 cells were then seeded onto each coverslip and allowed to attach overnight at 37 °C with 5% CO_2_. Cells were then washed with PBS.

A 5 μm spherical silica microsphere (polysciences) was mounted to the tip of a PNP-TR-TL cantilever (NanoWorld) with UV-curable glue. The tip was then loaded onto a liquid perfusion cantilever holder. The cell samples with PBS were loaded onto the 37 °C environmental chamber on a Cypher ES AFM (Asylum Research). Contact mode was used to obtain the force curves. To confirm cell attachment and compatibility with the system, a different tip, BL-AC40TS-C2 (Olympus), was used to image the cells. GetReal™ was used to calibrate the tip spring constant and the inverse of the optical lever sensitivity (InvOLS), using the thermal method. The relative trigger point voltage was set to be 0.1 V, and the indentation depth was set to be 200 nm. 5–10 force curves per cell were obtained on cell periphery. All measurements were completed within 30 min after the placement of the sample. The Hertz model $E= \frac{3\left(1-{\upnu }^{2}\right)F}{4\sqrt{R {I}^{3}}}$ where E, elastic modulus; F, force; R, sphere radius; I, indentation, and ν, Poisson ratio of cell, was fitted to the approaching force curve to extract the apparent elastic modulus.

### Cell morphology

Cells were seeded on collagen I-coated coverslips as described above (5,000 cells per 12 mm diameter coverslips). After 12 h incubation, cells were washed with PBS, stained with rhodamine-phalloidin and imaged by confocal fluorescence microscopy (Leica TCS SP5 Spectral confocal microscope and Volocity software (Perkin Elmer)).

### Rac-1 pull-down assay

Rac-1 Pull-down assay was performed according to Rac1/cdc42 activation assay instructions (EMD Millipore). Briefly, 4 million primary fibroblasts were starved overnight and stimulated with 10% FBS for 10 min prior to being lysed in 500 μl of Mg2^+^ lysis buffer. PAK-1 PBD agarose beads were incubated with the cell lysates to capture GTP-bound Rac1. Western blot was used to visualize Rac1 pull-down results.

### Western blot

Cell lysates were extracted using RIPA buffer supplemented with cOmplete EDTA-free protease inhibitor cocktail (Roche), 1 mM sodium fluoride, and 1 mM sodium orthovanadate. Protein concentration was determined using BCA protein assay kit according to supplier’s instructions (Thermofisher). Standard western was then performed, and the Licor Odyssy CLx system was used to visualize blots. Densitometry analysis was performed using ImageJ Gels plugin.

### Image analysis

For fluorescence intensity analysis, background was subtracted using the “rolling ball” algorithm. Circularity was defined as $\frac{4\pi *Cell \,\,Area}{{Cell \,\,Perimeter}^{2}}$. Directionality and colocalization test plugins on ImageJ were used to analyze F-actin and pixel-to-pixel correlation between actin and WAVE2 signals respectively.

### Reagents

The following primary antibodies were used for IF: anti-fibronectin (1:1000, Abcam, ab2413), anti-mouse collagen type I (1:1000, EMD Millipore, AB765P), anti-collagen VI (1:100, Abcam, ab6588), anti-WAVE2 (1:50, Cell Signaling, ab3569S), anti-ARPC2 (1:50, Abcam, ab11798), anti-Rac1 (1:100, Abcam, ab155938).

The following primary antibodies were used for flow cytometry: Biotin anti-mouse CD29/β1 (1:250, BioLegend, 102203), Biotin Armenian Hamster IgG Isotype Ctrl (1:250, BioLegend, 400903). Streptavidin-APC (1:50, Thermo Fisher, SA1005) was used as the secondary.

The following primary antibodies were used for western blot: anti-WAVE2 (1:1000, Cell Signaling, ab3569S), anti-ARPC2 (1:1000, Abcam, ab11798), anti-Rac1 (1:1000, Millipore, 05-389), anti-β-actin (1:1000, Abcam, ab8226).

The following secondary antibodies were used for immunofluorescence staining: anti-rabbit IgG Alexa Fluor 488 (1:1000, Invitrogen, a11008), anti-goat IgG Alexa Fluor 488 (1:1000, Invitrogen, a21467). The following secondary antibodies were used in compatible with the Licor Odyssy DLx system: anti-mouse IgG Alexa Fluor 680 (1:1000, Invitrogen, a10038), anti-rabbit IgG Alexa Fluor 800 (1:1000, Invitrogen, a32735).

### Statistical analyses

Error bars represent standard deviation (SD) unless stated otherwise. An unpaired student’s t-test was used when comparing two groups. For comparisons between three or four groups, one-way analysis of variance (ANOVA) tests with Tukey’s post hoc tests and multiple comparisons were utilized. For comparisons between three or four groups with time component (time lapse studies), two-way ANOVA tests using mixed-effects model with Geisser-Greenhouse correction followed by Sidak’s multiple comparison test were utilized. All statistical analyses were performed using GraphPad Prism 9.

### Ethical approval

All animal procedures were performed in accordance with institutional guidelines and based on protocol (2019-10867) approved by the Yale Institutional Animal Care and Use Committee (IACUC).

## Results

### Diabetes affects dermal fibroblast shape and F-actin directionality

**Figure 1:**
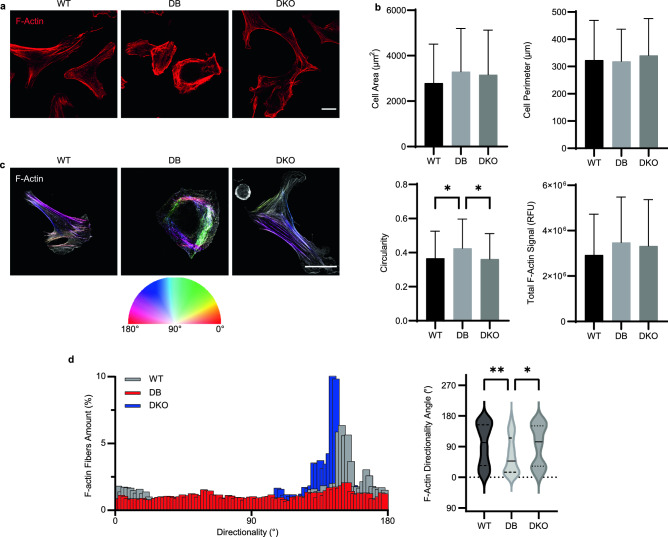
DB primary dermal fibroblasts exhibit rounded morphology. (**a**) Confocal images of WT, DB, and DKO dermal fibroblasts stained for F-actin (rhodamine phalloidin) show differences in cell shape. Scale bar is 20 μm. (**b**) Quantifications of cell area, perimeter, circularity, and total F-actin signals show rounded morphology of DB dermal fibroblasts compared to WT and DKO cells while maintaining a similar level of actin expression. One-way ANOVA followed by Tukey’s post hoc test and multiple comparison test (n_animals_ = 3 per genotype, n_cells_ = 30 per animal). *p < 0.05. (**c**) Representative grey scale images of F-actin overlayed with fiber direction show less directed F-actin bundles in DB cells compared to WT and DKO. Scale bar is 40 μm. **d** Representative histograms of fiber directionality across three groups. Measurements of F-actin directionality angle reveal lower directionality angles in DB dermal fibroblasts. One-way ANOVA followed by Tukey’s post hoc test and multiple comparison test (n_animals_ = 3 per genotype, n_cells_ = 20 per animal). **p < 0.01, *p < 0.05. – represents mean and -- represents the 25th and 75th percentiles.

Primary dermal fibroblasts from WT, DB, and DKO mice were isolated, stained with rhodamine-phalloidin to visualize F-actin, and examined with confocal microscopy (Fig. 1a). Image analysis revealed that DB fibroblasts had more circular morphology than WT or DKO cells even though cell area and perimeter were comparable (Fig. 1b). In addition, F-actin in DB cells was concentrated near the cell periphery, and stress fibers were less prominent and shorter in length. Since the intracellular actin network was comprised of stress fibers and cortical actin, our observations suggested that DB fibroblasts might experience a dynamic shift from stress fibers to cortical actin. In contrast, WT fibroblasts displayed spindle-shaped morphology that is conventionally observed in fibroblast cell lines, and is associated with their migratory behavior (Fig. 1a). Despite changes in subcellular distribution, image analysis revealed similar levels of actin signal (Fig. 1b).

Next, F-actin organization was investigated by directionality analysis. Remarkably, F-actin bundles were randomly pointed inside DB cells, whereas in WT cells they displayed increased directionality (Fig. 1c). F-actin fiber directionality was measured to confirm these observations. Figure 1d showed representative histograms of F-actin bundles in DB fibroblast with even distribution of directionality angles, whereas those in WT and DKO cells had bell-shaped distributions. Quantification of average directionality angles showed a closer-to-zero mean in DB cells, which was less than the other two groups (Fig. 1d). These observations highlight the randomness of actin bundle arrangements in DB fibroblasts, which may have serious implications in functional outputs, such as force generation and migration. Importantly, the morphology and actin directionality of diabetic dermal fibroblasts deficient in TSP2 (DKO) closely resembled those of WT cells, suggesting an association between TSP2 expression and cytoskeletal organization.

### Diabetic ECM derived from fibroblasts have thickened fibrils and hinder fibroblast transmigration

**Figure 2:**
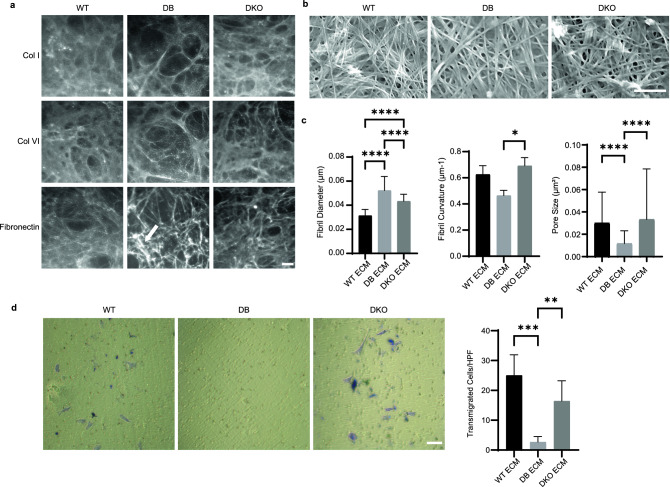
Cell-derived ECM from diabetic fibroblasts have thickened fibrils and lower porosity that hinder fibroblast transmigration. (**a**) IF images of ECM derived from WT, DB, and DKO fibroblasts show ECM proteins (collagen type I, collagen type VI, and fibronectin) organization. Arrow highlights the fibronectin-enriched matrix in ECM derived from diabetic fibroblasts. Scale bar is 40 μm. (**b**) Representative scanning electron images of ECM show thickened fibrils on a microscopic scale. Scale bar is 1 μm. (**c**) Left, measurements of fibril diameter show increased fibril size in diabetic ECM. Data were collected by averaging the diameter from 5 random locations along a fibril (n_animals_ = 3 per group, n_fibrils_ = 30 per animal). Middle, measurements of fibril curvature show increased curvature in DKO ECM compared to DB ECM (n_animals_ = 3 per genotype, n_fibrils_ = 60 per animal). Error bar refers to standard error of mean. Right, measurements of pore size show decreased size in DB ECM (n_animals_ = 3 per genotype, n_pores_ = 60 per animal). One-way ANOVA followed by Tukey’s post hoc test and multiple comparison test. ****p < 0.0001, *p < 0.05. (**d**) Left, representative images of the bottom fields of transwells after 8 h show fewer transmigrated cells in group with DB ECM. Cells were stained purple. Scale bar = 100 μm. Right, quantification of transmigrated cells after 8 h show fewer cells transmigrated through DB ECM compared to WT and DKO ECM. Data were collected by averaging cell numbers from 5 random fields per sample (n_animals_ ≥ 4 per group). One-way ANOVA followed by Tukey’s post hoc test and multiple comparison test. ***p < 0.001, **p < 0.01.

Fibroblasts play a major role in the deposition and remodeling of ECM. Diabetic fibroblasts derived from KK strain mice^35^ and diabetic patients^36^ have been reported to have altered biosynthesis of ECM protein. To investigate whether ECM production was also affected in db/db model, we analyzed the deposition of collagen type I, type VI, and fibronectin in cell-derived ECM by immunocytochemistry. Phenotypically, we observed fibronectin-enriched fibers in diabetic ECM, while the deposition of different collagen fibers appeared similar (Fig. 2a). SEM analysis was performed to explore fibril formation in greater detail and confirmed the increased fibril thickness in diabetic ECM (Fig. 2b, c). Specifically, quantification of fibril diameter showed that they increased in both DB and DKO ECM compared to WT ECM. However, DKO fibrils were thinner than DB fibrils and more curved, indicating distinctive differences between the two groups. Finally, ECM pore size, defined as circular area between fibrils, was quantified, and it was found to be smaller in DB ECM. Taken together, these observations show that DB ECM is characterized by distinct differences including deposition of thicker and less curved fibrils forming a network with smaller pore size. Notably, DKO ECM deposition was closer to that observed in WT.

ECM does not only provide structural support for cell growth, but also influences cell functions through mechano-sensing and other mechanisms^37^. To probe this aspect, cell migration through WT, DB, or DKO ECM was measured in a modified assay. Specifically, we utilized a migration assay where the three different cell types were cultured on the top chambers of transwells, allowed to deposit ECM, and then decellularized to produce a layer of cell-derived ECM. NIH/3T3 were then evaluated for their ability to migrate over 8 h by counting the number of cells that reached the bottom of the transwell (Fig. 2d). Quantification revealed the number of cells that migrated through DB ECM was significantly smaller than that through WT ECMs, whereas the number of cells that migrated through WT and DKO ECM were similar (Fig. 2d). Based on the structural analysis of ECM, it is likely that the smaller pore size and the nature of fibrils in DB ECM contribute to reduced migration of NIH/3T3 cells.

### Diabetic fibroblasts exhibit impaired force generation on 2D and in 3D collagen matrix

**Figure 3:**
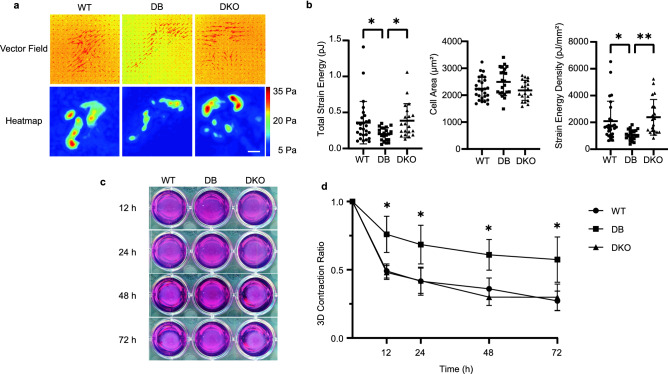
Diabetic fibroblasts generate less force on 2D and 3D collagen matrix. (**a**) Top, representative vector fields of WT, DB, and DKO fibroblasts seeded on 12 kPa polyacrylamide gels show the underlying beads displacement. Bottom, corresponding heatmaps of traction force show the magnitude and distribution of stress. Scale bar = 40 μm. (**b**) Left, measurements of total strain energy for each group show the lack of strain energy in DB cells. Middle, cell area measurements show no differences among the groups. Right, strain energy density measurements show the lack of force generation per unit area in DB fibroblast (n_animals_ = 3 per genotype, n_cells_ ≥ 20 per animal). One-way ANOVA followed by Tukey’s post hoc test and multiple comparison test. **p < 0.01, *p < 0.05. (**c**) Representative images of 3D collagen gel contraction over 72 h show impaired 3D contraction of DB fibroblasts. (**d**) Contraction ratios measured over 72 h show significant contraction among WT and DKO compared to DB fibroblasts. Contraction ratios were calculated by dividing gel size by the starting size. Data were collected by averaging 3 gels per group (n_animals_ ≥ 4 per genotype). Two-way ANOVA using Mixed-effects model with Geisser-Greenhouse correction followed by Sidak’s multiple comparison test. *p < 0.05.

The success of wound closure heavily depends on viable fibroblasts to perform their mechanical functions^38^. Traction force and 3D contractile force generated by fibroblasts are essential to their functions as migratory and contractile cells respectively^39^. Therefore, we investigated force generation of dermal fibroblasts using TFM and 3D gel contraction assay. WT, DB, or DKO fibroblasts were seeded on top of 12 kPa polyacrylamide gels conjugated with type I collagen. By using confocal microscopy to track the underlying particle movements between force-loaded and cell-free images, we were able to construct vector field and heatmaps of traction force (Fig. 3a). By aggregating the strains of each cell, we found that DB fibroblasts exerted significantly less strain energy (0.1986 ± 0.08698 pJ) compared to WT (0.3555 ± 0.2907 pJ). By dividing the strain energy by the area of each cell, not surprisingly, we also discovered that DB fibroblasts exerted less strain energy per unit area (1075 ± 380.6 pJ/mm^2^) than WT (2096 ± 1484 pJ/mm^2^) (Fig. 3b). Despite these defects, DB cells had similar overall cell area. These results indicated that under diabetic conditions, fibroblasts were not able to generate as much strain energy on a 2D soft substrate compared to WT. In contrast, DKO fibroblasts were able to generate as much strain energy (0.3822 ± 0.2343 pJ) and per unit area (2385 ± 1327 pJ/mm^2^) as their WT counterparts. This suggests that, despite diabetic conditions, DKO cells are not impaired in strain energy generation.

In addition to measuring forces on a 2D surface, the ability of cells to contract in a 3D matrix was evaluated (Fig. 3c). Specifically, WT, DB, or DKO fibroblasts were cultured within a collagen gel and allowed to contract for 72 h. By using contraction ratio as a proxy of contractility, defined as $\frac{Area \,\,of \,\,gel}{Area \,\,of \,\,original \,\,gel}$, we found that DB fibroblasts were dysfunctional compared to WT or DKO for a period of 72 h when contraction reaches maximal level (Fig. 3d). MTT assays in all cell types indicated that the rate of cellular proliferation did not differ and did not influence contractility (Fig. S1a). Moreover, we determined the surface expression of β1 integrin subunits and found it to be similar among the three groups, eliminating the possibility of differential β1 integrin binding to the collagen matrix as a contributor to reduced contraction (Fig. S1c). Taken together, the reduced traction force generation and gel contraction imply defects in cytoskeletal function in DB cells, and this could be reflected in irregular organization, fewer stress fibers, and less directionality of actin fibers (Fig. 1).

### Diabetic fibroblasts show slow migration and increased cell stiffness

**Figure 4:**
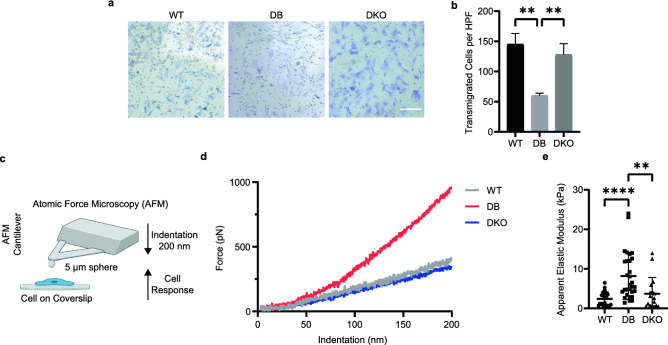
Diabetic fibroblasts have impaired migration and increased stiffness. (**a**) Representative IM images of transwell migration assay show fewer DB fibroblasts transmigrated after 24 h. Cells were stained purple. Scale bar = 200 μm. (**b**) Cell numbers per high power frame (HPF 20X objective) show fewer DB fibroblast transmigration. Data were collected by averaging 3 HPFs (n_animals_ = 3 per genotype). One-way ANOVA followed by Tukey’s post hoc test and multiple comparison test. ***p < 0.001. (**c**) schematic (created with BioRender.com) shows an AFM test using a spherical tip. Image is not up to scale. (**d**) Representative force curves show differences during indentation. (**e**) Apparent elastic moduli calculated from Hertz Model show increased stiffness in DB fibroblasts. Data were collected by averaging 5 force curves per cell (n_animals_ = 3 per genotype, n_cells_ ≥ 6 per animal). One-way ANOVA followed by Tukey’s post hoc test and multiple comparison test. ****p < 0.0001. **p < 0.01.

Standard transwell migration assay was used to study the migratory ability of WT, DB, and DKO cells. After 24 h of migration in a serum gradient, fewer DB fibroblasts appeared to migrate in the transwell assay, and this was confirmed by quantification of stained membranes of transmigrated cells (Fig. 4a, b). In contrast to DB cells, a similar number of DKO fibroblasts migrated as WT.

Because the rate of migration is often correlated with cell stiffness^40^, AFM was utilized to probe the elastic modulus of the cells (Fig. 4c). We were able to image cells seeded on collagen-coated coverslip (Fig. S2b) using a fast scan compatible tip (Fig. S2a). By using a custom-made spherical probe (Fig. S2c), we were able to perform indentation experiments on the cell periphery from which we extracted the information on cell stiffness (Fig. S2d, e)^41,42^. Figure 4d shows representative force curves during indentation. DB fibroblast exhibited higher force compared to WT (Fig. 4d). Since the experimental set-up satisfied small strain and small contact requirement, with isotropic and elastic body assumption, we applied Hertz model to the approaching force curve and obtained the apparent elastic modulus for each cell. DB fibroblasts exhibited increased stiffness (8.180 ± 5.904 kPa) compared to WT (2.419 ± 1.711 kPa) (Fig. 4e). These results demonstrate the increased stiffness of DB fibroblasts, which was associated with decreased migratory ability based on the transwell assay. Importantly, under the same diabetic condition, DKO fibroblasts displayed normal migratory ability and stiffness (3.722 ± 4.082 kPa).

### Diabetic fibroblasts display disrupted lamellipodia associated with low Rac1 activity and irregular WAVE2 localization

**Figure 5:**
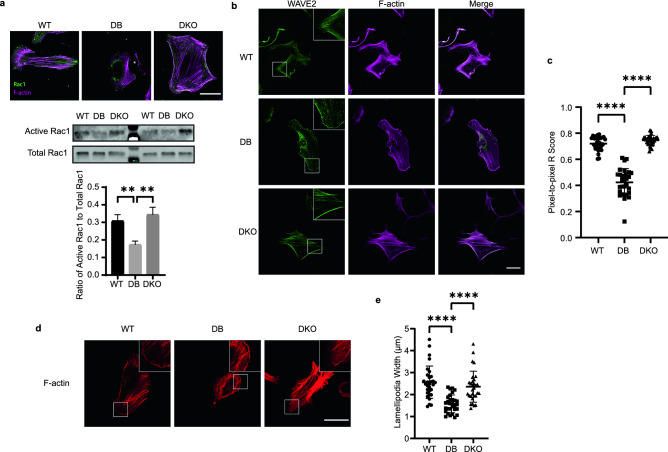
Dysfunction in diabetic fibroblasts is associated with lack of active Rac1 and WAVE2. (**a**) Top, representative IF images of Rac1 and F-actin show Rac1 localization in relation to F-actin. Scale bar = 40 μm. Middle, representative western blot of GTP-bound Rac1 and total Rac1 shows lack of active Rac1 in DB fibroblasts. Full blots are available in Supplementary Fig. 4. Bottom, densitometry analysis shows significant decrease of active Rac1 in DB when normalized to total Rac1 signal (n_animals_ = 3 per group). One-way ANOVA followed by Tukey’s post hoc test and multiple comparison test. *p < 0.05. (**b**) Representative IF images of dermal fibroblasts stained for WAVE2 and F-actin show co-localization of WAVE2 signals and F-actin. Scale bar = 40 μm. (**c**) Quantification of R score between WAVE2 signals and F-actin signals show differences across the three genotypes (n_animals_ = 3 per genotype, n_cells_ = 10 per animal). One-way ANOVA followed by Tukey’s post hoc test and multiple comparison test. ****p < 0.0001. (**d**) Representative IF images of dermal fibroblasts were stained for F-actin. Scale bar = 40 μm. (**e**) Measurements of lamellipodia width show decreased width in diabetic fibroblasts. Data were obtained by averaging lamellipodia width within each cell (n_animals_ = 3 per genotype, n_cells_ = 10 per animal). One-way ANOVA followed by Tukey’s post hoc test and multiple comparison test. ****p < 0.0001.

Rac1, a member of the Rho GTPase family, is known to drive major actin reorganization and induce lamellipodia formation by activating the WAVE2 complex^43^. Activated WAVE2 complex, in turn, associates with the Arp2/3 complex to enhance actin polymerization^44^. To explore whether this process was compromised in DB fibroblasts, the levels and distribution of total Rac1 as well as active Rac1 were determined by multiple approaches (Fig. 5a). Immunocytochemical detection of total Rac1^45^ in WT, DB, and DKO cells by confocal microscopy did not reveal significant differences in expression levels (Fig. 5a). However, its position in DB cells was primarily present in the center of the cell and did not co-localize with stress fibers, which differed from WT and DKO cells, suggesting that there was a lack of activated form of Rac1 in DB cells^46^. To further measure activation, a pull-down assay for active Rac1 (Rac1-GTP) was performed and showed a significant reduction in DB cell lysates and this was confirmed by densitometric analysis of the active Rac1 signal to total Rac1 (Fig. 5a). Because of the distinct subcellular distribution of Rac1 in DB cells, immunocytochemistry was performed to detect the subcellular distribution of WAVE2^47^. Confocal microscopy revealed that WAVE2 was found to be localized to the cell periphery and co-localized with F-actin fibers and in WT and DKO cells, as illustrated by the increase of white pixels in merged images (Fig. 5b). In contrast, in DB cells WAVE2 displayed a punctate deposition pattern and did not exhibit the same co-localization seen in the other cells, and this was analyzed by determining the R-score of pixel-to-pixel matching that revealed a significant decrease in correlation (Fig. 5c). However, the lack of co-localization between WAVE2 and F-actin did not affect the localization of Arp2/3 complex to the actin bundles (Fig. S3a). Moreover, western blot analysis revealed no difference in WAVE2 expression among the three genotypes, suggesting that the irregular distribution was not due to changes in levels (Fig. S3b).

Because WAVE2 and actin polymerization can directly influence lamellipodia formation, we analyzed their morphology in cells stained with rhodamine phalloidin (Fig. 5d). Specifically, the width of individual lamellipodia was determined form confocal images and found to be smaller in DB cells (Fig. 5d, e). This observation suggests that reduced Rac1 activation and suboptimal WAVE2-actin fiber association in DB cells leads to reduced lamellipodia formation. Remarkably, Rac1 localization and activation, and the WAVE2 complex-stress fiber colocalization were normal in DKO cells, and these were associated with normal formation of lamellipodia, suggesting that TSP2 plays a key role in cytoskeleton remodeling.

## Discussion

Fibroblasts are instrumental to the wound healing process, especially in diabetes, but much of the attention is given to dysregulation of immune responses and complications due to chronic inflammation and infections. In contrast, less is known about biomechanical defects in diabetic fibroblasts. In this work, we analyze diabetic fibroblasts by using multiple advanced microscopy techniques and provide insights into the biomechanical defect that impairs functions.

In the context of diabetes-induced ECM modifications, the observation of thickened fibronectin-enriched fibrils deposited by DB cells is consistent with numerous studies that utilized diabetic fibroblasts^35,36^ or fibroblasts in high glucose treatment^48^. In addition, our group has discovered that LOX, a collagen/elastin cross-linking enzyme, is regulated by TSP2 level^22^. Specifically, loss of TSP2 level leads to lower amount of LOX and increased matrix solubility. Other studies have observed that LOX directly regulates collagen fibril diameters^49,50^. Thus, we think that the increased biosynthesis of fibronectin, and increased TSP2 level in DB fibroblasts could both contribute to the thickening of the fibrils we observed microscopically. On the other hand, the loss of TSP2 in DKO fibroblasts might increase the solubility of collagen leading to normalization of fibril diameters. The pore size in DB ECM was significantly smaller than WT and DKO. Moreover, we observed that ECM derived from DB fibroblasts retarded the migration NIH/3T3 fibroblast to a greater extent than WT or DKO.

In addition, we observed that mouse DB fibroblasts displayed round morphology, which is consistent with previous analyses of fibroblast isolated from human chronic diabetic wounds^51^. This similarity demonstrated the similarity between diabetic patients and the db/db model. We show that the irregular morphology seen in DB fibroblasts is associated with the loss of coordinated F-actin fiber alignment, implying that diabetes alters the organization of the cytoskeleton without affecting overall actin expression. In addition, actin cytoskeleton is a dynamic network that is comprised of cortical actin filaments and stress fibers^40^. Based on the F-actin signal location and the length and width of the F-actin filaments, we postulate that there is a shift from stress fibers towards cortical actin in DB fibroblasts. In turn, this could lead to changes in cell shape and downstream mechanical outputs. Because cell shape strongly correlates with many cellular functions such as migration and force generation, this deregulation could cause the dysfunctional state of the diabetic fibroblasts.

Previously, diabetic erythrocyte^52^ and cardiomyocytes^53^ were reported to increase their stiffness in diabetes while coronary microvascular smooth muscle cells (CMSMC) had decreased stiffness^54^ when measured by AFM. Our report is the first to investigate the elastic modulus of diabetic dermal fibroblasts. In line with the discovery in erythrocytes and cardiomyocytes, we also found that dermal fibroblasts in diabetic conditions have increased stiffness. Both our study and the Trask group’s study of CMSMCs^54^, probed 200 nm into the cell and thus, measured mainly the cortical actin network stiffness. Despite using the same approach, unlike diabetic CMSMC, DB fibroblasts had increased stiffness suggesting that diabetes-induced changes are cell type-specific.

Contradictory to previous studies that suggest the positive correlation between traction force and stiffness in mouse embryonic fibroblasts (MEF), epithelial cells (MCF-10A)^55^, human alveolar epithelial cells (CCL-185)^56^, and human primary airway smooth muscle cells^57^, we report that in primary DB fibroblasts, traction force on soft substrate (12 kPa) is not positively correlated with stiffness. In the studies mentioned above, a single genotype was used, and the traction force-stiffness correlation was derived by either altering the stiffness using pharmacological treatment to stimulate actomyosin contractility^56,57^ or retrospectively correlating the stiffness to traction force after measurement^55^. However, our study used 3 distinct cell populations from primary sources without treatments. Similarly, another independent study that investigated traction force and stiffness in diabetic CMSMC from primary sources also arrived at the same conclusion as ours^54^. In addition, finite element modeling of varying cell stiffness also suggests that stiffening can lead to decreased traction force^58^. Taken together, these findings imply that the explanation behind stiffness and traction force, especially under various physiological conditions, is more complex than previously thought.

Primary dermal fibroblasts have previously been reported to have defective 3D contraction in collagen gel matrix under high-glucose treatment^59^, in line with the impaired 3D contractility we observed in DB fibroblasts. Our group has previously published impaired contraction of TSP2KO fibroblasts in collagen matrix, but the exact mechanism has not been elucidated^60^. However, depletion of TSP2 in the diabetic background resulted in improved 3D contractility. Perhaps this reflects the significant impact of supraphysiological TSP2 levels on DB fibroblasts. Additionally, combined with 2D traction force, we speculate that DB fibroblasts, due to high TSP2, have stiff actin cortex that is less pliable and more resistant to contraction.

Migration is a highly coordinated process that requires a dynamic shift of traction force between the front and the rear of a cell^61^. The relationship between cell migration speed and traction force is highly complex and depending on the primary mode of migration. The relationship between speed and traction force could be either positively or negatively associated. “Sliding” migration with small transient traction force at adhesion sites has been reported in multiple highly mobile cell types^62,63^. On the other hand, lamellipodia-mediated migration is more commonly observed in fibroblasts with strong focal adhesion and traction force^64,65^. Here, migration is evaluated as the rate of movement through collagen-coated pores against serum gradient. Similar surface expression of β1 integrin among the 3 genotypes suggests that there is adequate adhesion of DB fibroblasts to ECM. Thus, we believe that the weak traction force observed on 2D surface is relevant to the compromised migration of DB cells. Moreover, there is a long understanding in the cancer field that cell stiffness is negatively correlated with motility^40,66,67^. Consistent with this negative correlation, the increased stiffness of DB fibroblasts is shown to be associated with decreased migration. It is possible that the softness of their cell bodies aids WT and DKO fibroblasts to modify their shape and migrate through pores.

Based on the morphological and functional defects in DB fibroblasts, we probed a Rac1-dependent mechanism as a central link to cytoskeletal abnormalities. Rac1 belongs to Rho GTPase family and is known to be a central signaling node for lamellipodia formation and cell migration^68^. WAVE2 is regulated by GTP-bound Rac1 via IRSp53, a substrate for insulin receptor^43,69^ and in turn, regulates the actin cytoskeleton through the activation of the Arp2/3 complex via the verprolin central acidic (VCA) domain^44^. In addition, studies have shown that WAVE2 induces actin polymerization and lamellipodia formation, and deletion or disruption of WAVE2 strongly diminishes cell migration^44,70^. In the present study, Rac1 cytoplasmic localization was irregular, and the active form of Rac1 was found to be diminished in DB fibroblasts. We speculate that the decreased level of active Rac1 led to insufficient activation of WAVE2, which resulted in the decreased co-localization of WAVE2 complex with F-actin and Arp2/3. The lack of WAVE2 co-localization could downregulate the levels of actin branching and lamellipodia formation, which might explain the slower migration observed in DB fibroblasts. However, when TSP2 was depleted, active Rac1 returned to normal level, and WAVE2 complex co-localized with active actin polymerization sites, rescuing the impaired lamellipodia formation. The observed changes in active Rac1 levels are consistent with previous observations in non-diabetic Akt1 KO (TSP2 over-expressors) and Akt1/TSP2 DKO fibroblasts that have decreased and normal levels, respectively^21^. Importantly, the present study highlights the negative impact of insufficient level of active Rac1 and WAVE2-actin interactions on the biomechanical properties of DB cells. They also shed light on the previously undiscovered role of TSP2 in regulating cytoskeleton organization via Rac1-WAVE2 axis.

Collectively, our findings show that diabetic fibroblasts display multiple functional defects that are linked to irregular cytoskeletal organization and function. This is due, in part, to the presence of supraphysiological levels of TSP2 that are associated with decreased active Rac-1 and disruption of the Rac1-WAVE-2-actin axis. Even though the impact of other metabolic effects cannot be discounted, it is intriguing to consider that the cellular and molecular defects are corrected in DKO cells despite their diabetic status. Consistent with this observation, a previous study described restoration of delayed wound in db/db mice following depletion of TSP2^30^. Therefore, we conclude that some aspects of fibroblast dysfunction and tissue repair in diabetes can be rescued independent of metabolic status.

## Supplementary Information

Supplementary Figures.

## Data Availability

All data generated and analyzed in this study are included in this published article and its supplementary information.
