# Author Correction: Dysregulation of TSP2-Rac1-WAVE2 axis in diabetic cells leads to cytoskeletal disorganization, increased cell stiffness, and dysfunction

**DOI:** 10.1038/s41598-023-31191-w

**Published:** 2023-03-14

**Authors:** Hao Xing, Yaqing Huang, Britta H. Kunkemoeller, Peter J. Dahl, Ohvia Muraleetharan, Nikhil S. Malvankar, Michael P. Murrell, Themis R. Kyriakides

**Affiliations:** 1 Department of Biomedical Engineering, Yale University, New Haven, USA; 2 Department of Pathology, Yale University, New Haven, USA; 3 Department of Molecular Biophysics and Biochemistry, Yale University, New Haven, USA; 4 Department of Physics, Yale University, New Haven, USA; 5 Systems Biology Institute, Yale University, New Haven, USA; 6 Microbial Sciences Institute, Yale University, New Haven, USA; 7 Vascular Biology and Therapeutics Program, Yale University, New Haven, USA

Correction to: *Scientific Reports* 10.1038/s41598-022-26337-1, published online 28 December 2022

The Funding section in the original version of this Article was incomplete.

“This works was funded by National Institutes of Health (Grant nos. DK115969 and 1DP2AI138259-01).”

now reads:

“This work was funded by National Institutes of Health (Grant nos. DK115969, DK132645 and 1DP2AI138259-01).”

The original Article has been corrected.

