# Supplementary material for: Dysregulation of TSP2-Rac1-WAVE2 axis in diabetic cells leads to cytoskeletal disorganization, increased cell stiffness, and dysfunction
Source: Sci Rep. 2022 Dec 28;12:22474. doi: 10.1038/s41598-022-26337-1 (PMC9797577; doi:10.1038/s41598-022-26337-1)
Supplement: Supplementary file 1 — Supplementary Figures. [file 41598_2022_26337_MOESM1_ESM.docx]

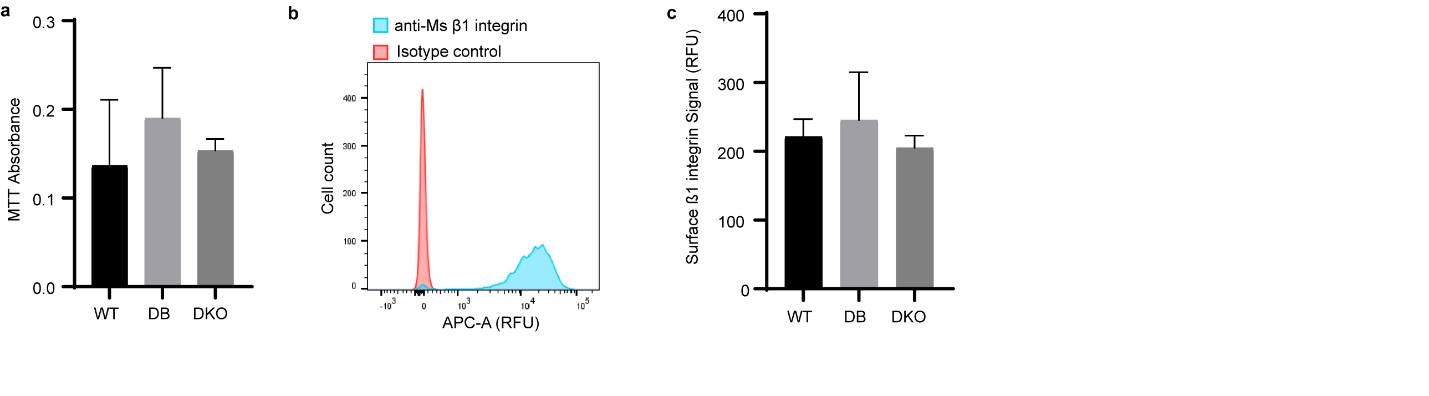


**Fig. S1 Proliferation assay and β1 integrin subunit surface expression show no difference among the groups. a** MTT assay performed on 10,000 primary fibroblasts seeded in 96 well plate showed no difference in proliferation speed. n≥4 animals. No statistical significances were discovered using One-way ANOVA test. **b** Strong separation of populations of cells stained with anti-Ms β1 integrin antibody and isotype control antibody demonstrated antibody specificity. **c** β1 integrin subunit surface expression was measured by flow cytometry. n≥4 animals. No statistical significances were discovered using One-way ANOVA test.


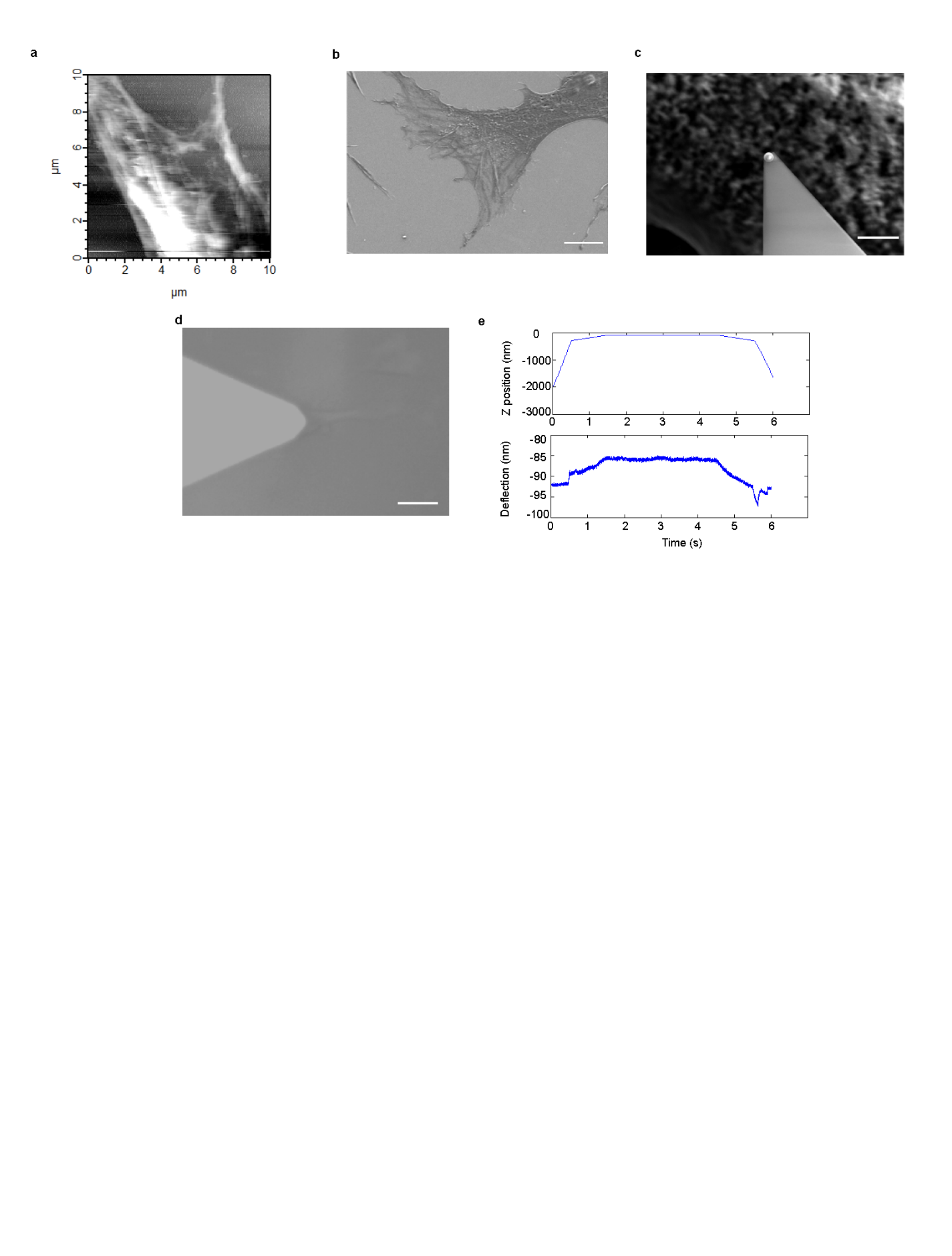


**Fig. S2 AFM can successfully target cell periphery. a** height channel image of fibroblast periphery was obtained using a BL-AC40TS-C2 tip and fast force mode under the same experimental condition. **b** SEM image of fibroblasts seeded on collagen I-coated coverslips show normal cell attachment and spreading. Scale bar = 10 μm. **c** SEM image of custom-made PNP-TR-TL-50 tip shows the position and mounting of 5 μm bead. Scale bar = 20 μm. **d** Field of view shows the position of tip in relation to underlying cell sample. Scale bar = 100 μm. **e** Top, Z-position graph shows the approaching (1 s), hold (3 s), and retraction (1 s) during stiffness measurement. Bottom, representative deflection shows the deflection corresponding to the Z-position.


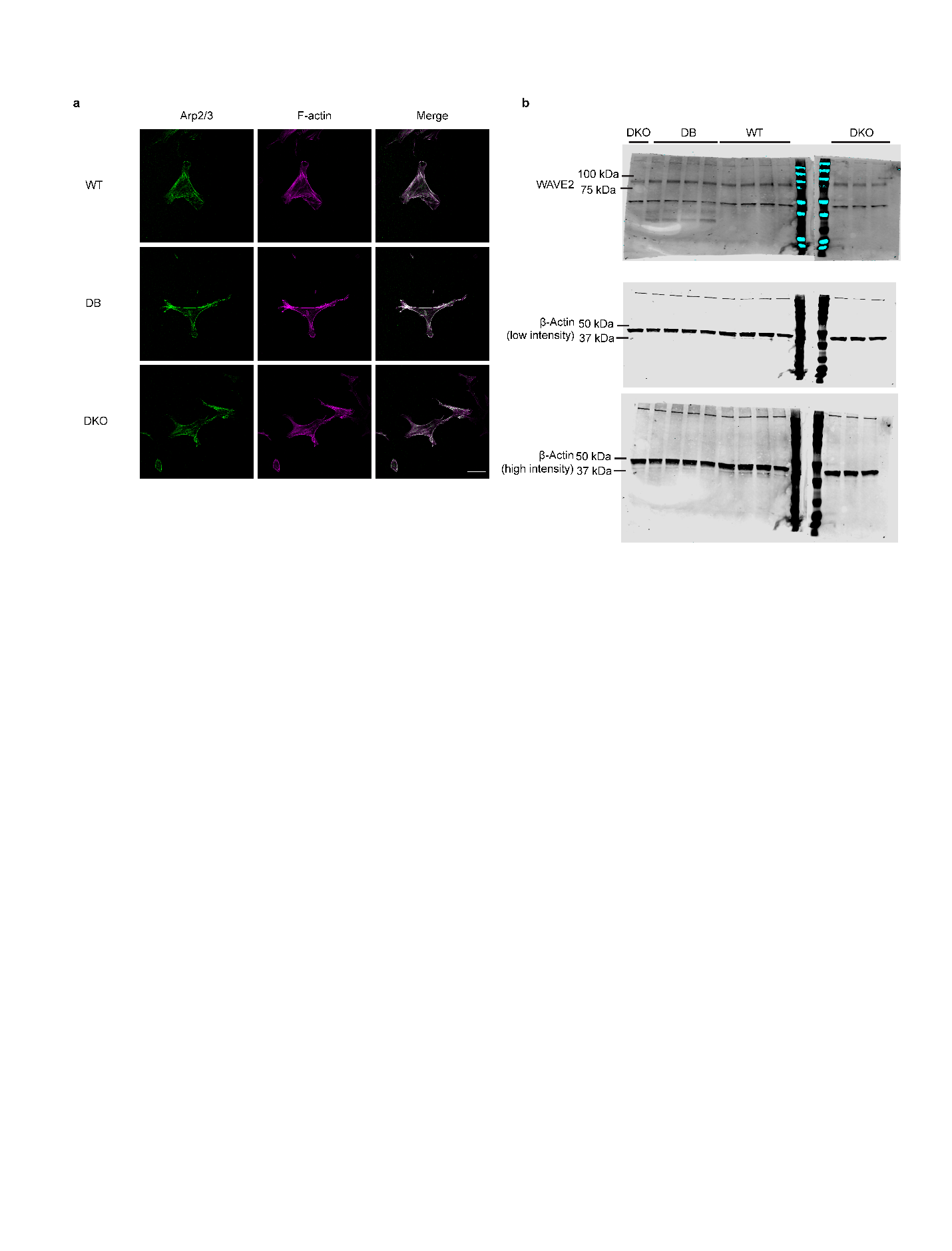


**Fig. S3 Arp2/3 colocalizes with F-actin in dermal fibroblasts. a** Representative IM images of dermal fibroblast stained for Arp2/3 subunit 2 and F-actin show localization of Arp2/3 signals in relation to the actin bundles. Scale bar = 40 μm. **b** Western blots show no differences in WAVE2 expression across groups. n = 4 animals per genotype.


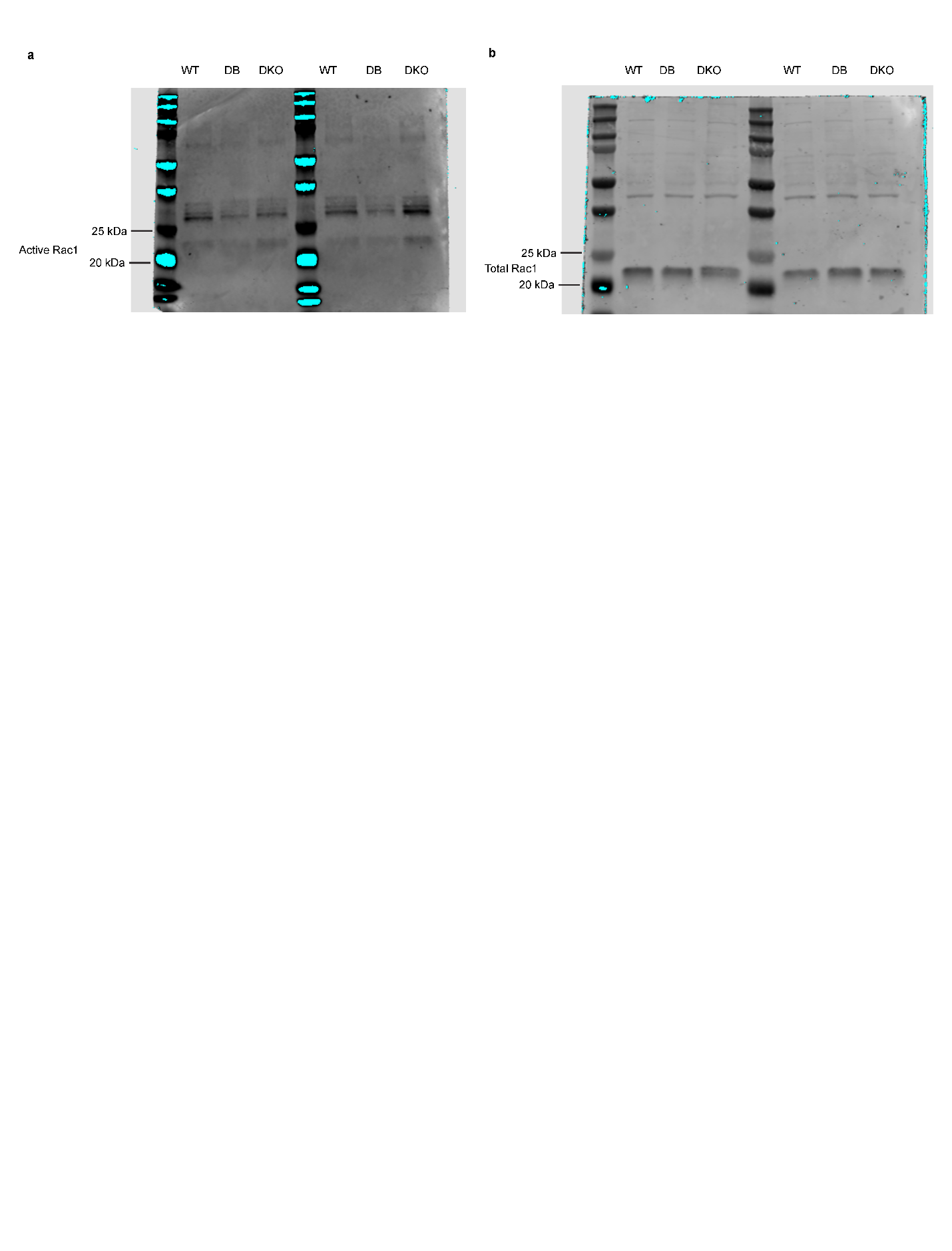


**Fig. S4 Uncropped western blots for active and total Rac1. a** Uncropped western blot of active Rac1 shown in Fig. 5 **b** Uncropped western blot of total Rac1 shown in Fig. 5
